# Factors Influencing the Perceived Effectiveness of COVID-19 Risk Assessment Mobile Application “MorChana” in Thailand: UTAUT2 Approach

**DOI:** 10.3390/ijerph19095643

**Published:** 2022-05-06

**Authors:** Nattakit Yuduang, Ardvin Kester S. Ong, Yogi Tri Prasetyo, Thanatorn Chuenyindee, Poonyawat Kusonwattana, Waranya Limpasart, Thaninrat Sittiwatethanasiri, Ma. Janice J. Gumasing, Josephine D. German, Reny Nadlifatin

**Affiliations:** 1 School of Industrial Engineering and Engineering Management, Mapúa University, 658 Muralla St., Intramuros, Manila 1002, Philippines; nuttakit33@gmail.com (N.Y.); aksong@mapua.edu.ph (A.K.S.O.); thanatorn_chu@rtaf.mi.th (T.C.); mr.k.poonyawat@gmail.com (P.K.); mjjgumasing@mapua.edu.ph (M.J.J.G.); jdgerman@mapua.edu.ph (J.D.G.); 2 School of Graduate Studies, Mapúa University, 658 Muralla St., Intramuros, Manila 1002, Philippines; 3 Department of Industrial Engineering and Aviation Management, Navaminda Kasatriyadhiraj Royal Air Force Academy, Bangkok 10220, Thailand; amarit@rtaf.mi.th; 4 Department of Chemistry, Faculty of Science, Mahidol University, Bangkok 10400, Thailand; l.waranya06@gmail.com; 5 Department of Information Systems, Institut Teknologi Sepuluh Nopember, Kampus ITS Sukolilo, Surabaya 60111, Indonesia; reny@its.ac.id

**Keywords:** COVID-19 contact tracing, UTAUT2, PMT, mobile application, MorChana

## Abstract

COVID-19 contact-tracing mobile applications have been some of the most important tools during the COVID-19 pandemic. One preventive measure that has been incorporated to help reduce the virus spread is the strict implementation of utilizing a COVID-19 tracing application, such as the MorChana mobile application of Thailand. This study aimed to evaluate the factors affecting the actual usage of the MorChana mobile application. Through the integration of Protection Motivation Theory (PMT) and Unified Theory of Acceptance and Use of Technology (UTAUT2), latent variables such as performance expectancy (PE), effort expectancy (EE), social influence (SI), facilitating conditions (FC), hedonic motivation (HM), habit (HB), perceived risk (PCR), self-efficacy (SEF), privacy (PR), trust (TR), and understanding COVID-19 (U) were considered to measure the intention to use MorChana (IU) and the actual usage (AU) of the mobile application. This study considered 907 anonymous participants who voluntarily answered an online self-administered survey collected via convenience sampling. The results show that IU presented the highest significant effect on AU, followed by HB, HM, PR, FC, U, SEF, PE, EE, TR, and SI. This is evident due to the strict implementation of using mobile applications upon entering any area of the vicinity. Moreover, PCR was not seen to be a significant latent factor affecting AU. This study is the first to have evaluated mobile contact tracing in Thailand. The integrated framework can be applied and extended to determine factors affecting COVID-19 tracing applications in other countries. Moreover, the findings of this study could be applied to other health-related mobile applications worldwide.

## 1. Introduction

COVID-19 contact-tracing mobile applications have been some of the most important tools during the COVID-19 pandemic [1,2]. Despite the protocols implemented worldwide, the effects of the COVID-19 pandemic have still been evident across multiple countries. Barouki et al. [3] explained how this pandemic has challenged the economics of the world, especially those of health systems. It was explained that much focus nowadays is on the response and development to reduce the spread of the virus [3,4]. One response action taken by most countries in Asia was the implementation of COVID-19 tracing applications available through mobile phones. In the Philippines, the COVID-19 tracing application is called StaySafe. Saudi Arabia has Tetamman, Tabaud, and Tawakkalna [5]. In addition, Zhou et al. [6] explained the different mobile applications, such as Alipay in China and Aarougya Setu in India. These COVID-19 mobile trackers have been widely utilized in their respective countries to help reduce the spread of the virus.

Mobile applications such as COVID-19 tracking applications have been considered in several studies. The study of Drew et al. [7] considered evaluating trackers from the United Kingdom and the USA. Their study presented how the application considered several factors such as risk, symptoms, and clinical outcomes. Noronha et al. [8] considered a tracker in Canada, while Ming et al. [9] considered a newly developed tracker in the USA. Abhinav et al. [10] investigated a tracker from India and [11] considered trackers from the United Kingdom, Canada, Brazil, France, Spain, Greece, Russia, Australia, USA, Turkey, Czech Republic, Ghana, Bahrain, Iceland, Hungary, New Zealand, and Norway. They presented how the different COVID-19 tracing mobile applications have been beneficial; however, some indicated necessary improvements in utility and usability. Moreover, Asian countries such as Bangladesh, India, Iran, Vietnam, China, Saudi Arabia, Israel, Singapore, and Malaysia were also considered. However, no studies were available for the contact-tracing application from Thailand, MorChana.

**Figure 1:**
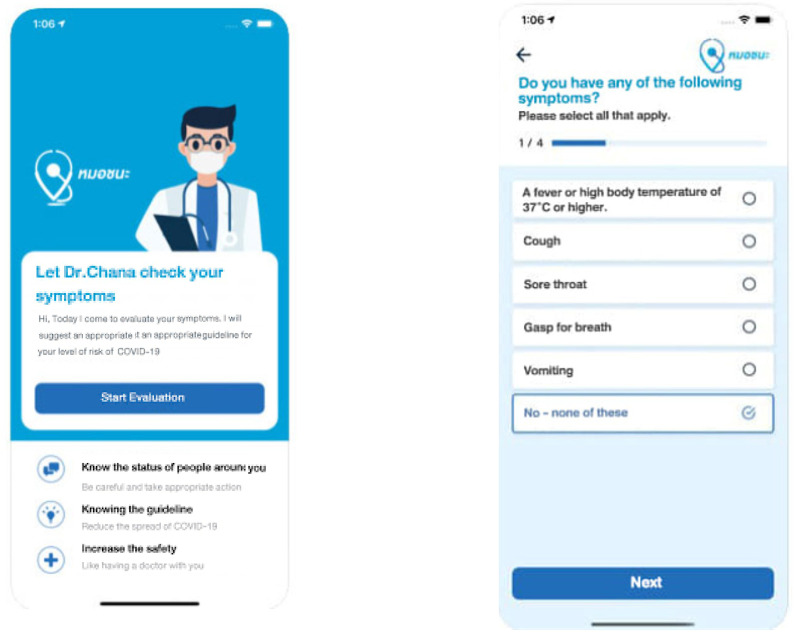

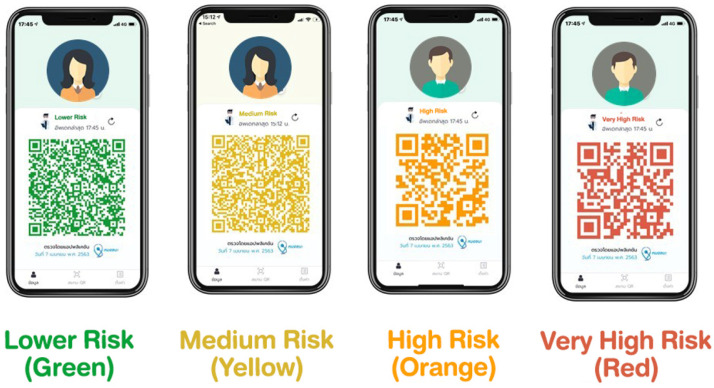
User interface of MorChana application.

MorChana, translated as Doctor Wins, is a COVID-19 tracing application utilized by Thailand. Presented in Figure 1 is the MorChana user interface, which is required before entering an area or vicinity in Thailand. The application provides a contact-tracking solution that allows mobile device users to self-assess and determine possible risk levels based on their contact and travel history with people. These data are collected in the database to trigger an alarm such as a push notification when users enter a high-risk area. After downloading MorChana from the Apple Store (IoS) or Play Store (Android), users can access the application during their trip and can immediately know from a notification from the application through the color-based indication if a high-risk user has been in the area in the last 14 days. This application was made available in April of 2020.

For MorChana, each person is given a QR code that health authorities can scan upon request. The color of the QR code reflects the risk level, which is calculated from questions about symptoms and travel history. Then, individuals are able to manage their actions appropriately by quarantining themselves for 14 days and closely monitoring their health for related COVID-19 symptoms. The utilization of MorChana is required when going to areas in Thailand, even for tourists. This new mobile application should be evaluated since studies have presented how forcing people to utilize an application does not necessarily have a positive effect [12]. Moreover, the application has helped Thailand create and strategize ways to reduce the spread [13]. In addition, Viwattanakulvanid [14] presented how the country was able to create plans such as testing; developed methods of communication to reduce risks, and positively manage the likely increase in spread; and helped the medical field through a reduction in cases. User expectations and high usability should be considered to have a positive effect on mitigation risk for health-related activities. Two popular theories widely utilized to model the acceptance of the new technology when it comes to health-related behaviors are the Unified Theory of Acceptance and Use of Technology (UTAUT2) and the Protection Motivation Theory (PMT).

UTAUT2 is a theoretical framework developed by Venkatesh et al. [15] that is widely used to analyze the user acceptance behavior of information technology products. Previously, there were several studies related to the usability of medical mobile applications. Alam et al. [16] used UTAUT2 to study the behavioral intentions of mHealth apps in Bangladesh. The proposed UTAUT2 model was extended to include other factors such as self-efficacy, privacy, trust, and lifestyle. It was seen that trust and hedonic motivation were the most significant influencing factors on the intention to use mHealth apps [16]. The behavioral intention to use Electronic Health Records (EHR) as an eHealth application in Egypt was investigated by Badran [17] through the UTAUT2 model. The results show that performance expectancy, price value, effort expectancy, and facilitating conditions significantly impact the usage intention of this new technology. Ware et al. [18] investigated the usage intention of the Medly app, and heart failure telemonitoring interventions on mobile phones in Toronto, Canada. UTAUT2 was used to explain the usage acceptance of this app. The results show that intention to use the Medly app was related to the UTAUT2 constructs of performance expectancy, effort expectancy, facilitation condition, social influence, and habit. In addition, UTAUT2 was integrated with PMT to analyze the user acceptance of personal health records in Malaysia [19]. It was shown how their model could holistically measure health-related behavior and technology acceptance. This study also considered the integration of UTAUT2 and PMT to measure the actual use of the MorChana COVID-19 tracing application.

PMT is a theory utilized to measure the coping and fear appraisal of people, usually utilized for health-related behaviors [20]. Kurata et al. [21] explained how PMT solely measures the perception of the risk and vulnerability of an individual. Studies such as that of van Bavel et al. [22] have considered utilizing PMT to determine the minimization of exposure and risk in order to enhance online security behavior. Moreover, Mousavi et al. [23] considered privacy protection on social networking sites utilizing PMT. Studies have shown that trust and privacy are significant factors affecting technology acceptance [22,23]. In addition, Yu et al. [24] integrated PMT and UTAUT2 to determine the factors affecting behavioral intention to use mobile health education. Their findings show how the model could be utilized for health-related behavioral studies. Recent research has tried to determine the intention to use COVID-19 contact-tracing applications, such as in Belgium [25], where Ezzaouia and Bulchand [26] collected 93 respondents from different countries, and Islam et al. [11] considered other countries’ COVID-19 contact-tracing applications. However, no studies were found dealing with COVID-19 tracing applications in Thailand, MorChana.

This study aimed to evaluate the intention to use the MorChana COVID-19 tracing application in Thailand. MorChana has been widely utilized in Thailand due to it being a requirement before entering any area or vicinity in the country. An integration of PMT and UTAUT2 was applied to measure Thai persons’ intention to use MorChana. Factors such as performance expectancy, effort expectancy, social influence, facilitating conditions, hedonic motivation, habit, perceived risk, self-efficacy, privacy, trust, and understanding COVID-19 were considered to measure intention to use MorChana, which would lead to measuring the actual use of the mobile application. This study is the first study to examine the behavioral intention to use the MorChana app in Thailand. The integrated framework can be applied and extended to determine the factors affecting COVID-19 tracing applications in other countries. Moreover, the findings of this study could be applied in other health-related mobile applications worldwide.

## 2. Conceptual Framework

**Figure 2:**
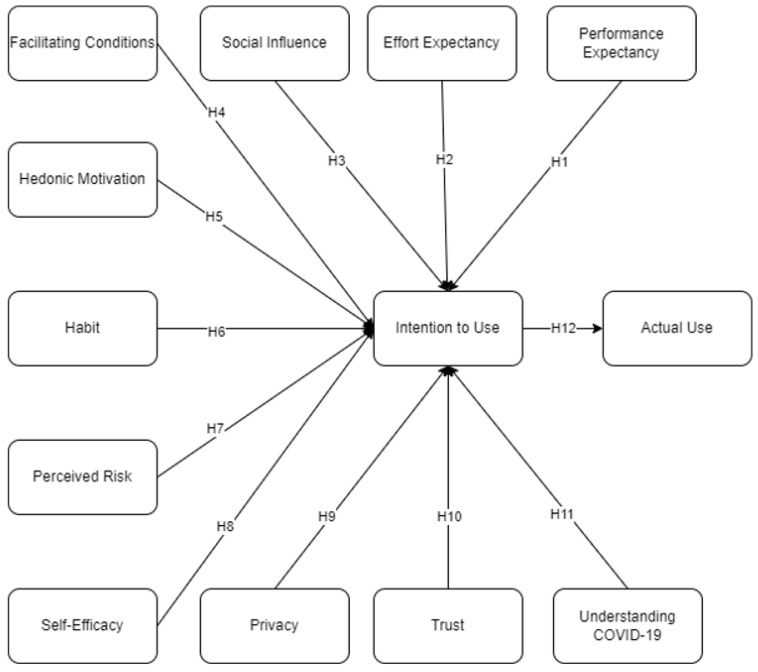
Conceptual framework.

Figure 2 represents the conceptual framework utilized in this study. The framework developed was through the integration of UTAUT2 and PMT [16,25,26] to measure the actual usage of the MorChana COVID-19 contact-tracing application in Thailand. Several factors such as performance expectancy (PE), effort expectancy (EE), social influence (SI), facilitating conditions (FC), hedonic motivation (HM), and habit (HB) from UTAUT2; and perceived risk (PCR), self-efficacy (SEF), privacy (PR), trust (TR), and understanding of COVID-19 (U) from PMT, together with intention to use (IU) and actual usage (AU), were considered in this study.

PE is defined as the way users perceive the benefits to them of a novel technology [27]. Alam et al. [16] found that PE has a significant impact on mobile service adoption. Applications that provide more utility are more likely to be used than apps that do not provide that utility. On the other hand, EE is defined as the degree of ease in using information systems [27]. Moreover, Venkatesh et al. [27] explained that SI is defined as a person’s belief that others think the technology should be used. Social influence is said to be highly essential in the early stages of a person’s experience with new technologies, because the knowledge obtained from the people around them would lead to the utility of a certain technology [16]. While its role gradually diminishes over time and eventually becomes insignificant as the technology persists, its experiences have provided a more practical basis for an individual’s continued use of the technology.

Due to the COVID-19 pandemic, mobile applications have recently been adopted to support COVID-19 risk management in many countries. Walrave et al. [25] used the UTAUT2 model to investigate the intention to use COVID-19 contact-tracing technology in Belgium. From their study, PE was the most influential factor, followed by FC and SI. Ezzaouia and Bulchand [26] studied the acceptance of contact-tracing apps from several countries using UTAUT2. The influencing factors of PE, FC, and SI were seen to be significant factors, together with other UTAUT2 variables. However, their study generalized the findings of several mobile applications. Therefore, it was hypothesized that:

**H1.**  *PE has a significant direct positive effect on intention to use the MorChana mobile application.*

**H2.**  *EE has a significant direct positive effect on intention to use the MorChana mobile application.*

**H3.**  *SI has a significant direct positive effect on intention to use the MorChana mobile application.*

FC refers to the context in which individuals are aware of organizational and technical infrastructure capabilities that facilitate the use of new information systems [27]. Previous studies on application acceptance have shown that users’ perceptions of convenience (i.e., having resources and support to use technology) directly influence behavioral intentions to use technology [28]. In addition, users who have better technical knowledge and skills in using apps are more likely to continue using them. Lallmahomed et al. [29] presented how FC is one of the most significant factors affecting intention to use technology. Thus, it was hypothesized that:

**H4.**  *FC has a significant direct positive effect on the intention to use the MorChana mobile application.*

HM is defined as the level of enjoyment derived from the use of innovative technology and is recognized as playing an essential role in the adoption and use of technology [27]. When clients perceive that the usage of new technology brings amusement, comfort, pleasure, and satisfaction, they will adopt that new technology. If a person feels that using a mobile application is satisfying, they are likely to use the mobile application [30]. Consequently, HB is described as the point at which people intend to continuously use an application and their continuous behavior would be based on learning or experience [27]. It is also considered a cognitive variable that reflects past experiences. When it is a daily activity for consumers to use health applications, it would lead to a positive significant effect on the usage intention [18]. It can also be said that initial usage intention is reactivated when consumers engage in health behavior, leading to positive repetition [31]. Habitual smartphone use has reached very high levels in developed countries such as the United States [32]. Amoroso et al. [32] found that satisfied customers are more likely to make use of a habit and consequently show a greater willingness to continue using these apps. Therefore, the following was hypothesized:

**H5.**  *HM has a significant direct positive effect on the intention to use a mobile application.*

**H6.**  *HB has a significant direct positive effect on the intention to use the MorChana mobile application.*

PCR is defined as a combination of uncertainty and the severity of associated consequences [20]. Risk is often associated with the perceived possibility of impact or loss under uncertain circumstances [21,33]. Reducing risk and increasing confidence will have a significant impact on behavioral acceptance and continuous patronage [34]. If people perceived the benefits of technology, then they would consider the acceptance of it. Therefore, it was hypothesized that:

**H7.**  *PCR has a significant direct positive effect on intention to use the MorChana app.*

SEF refers to a person’s technical skills or knowledge to accomplish tasks with electronic devices such as smartphones, and wireless technologies that motivate them to continue using them [20]. Previous studies have shown that self-efficacy influences willingness to use mobile applications and other eServices [35,36]. Thakur [36] explained how self-efficacy, together with satisfaction, would lead to the continuous intention to use a mobile application. Thus, it was hypothesized that:

**H8.**  *SEF has a significant direct positive effect on intention to use the MorChana mobile application.*

PR played a significant role in the successful implementation and adaptation of technology. It is defined as the extent to which a person’s individual right to control information about them by third parties is guaranteed [37,38]. The protection of user data is of great importance when using mobile applications for health-related information [39]. In addition, Mingxing et al. [39] explained how PR and TR are both significant factors affecting an individual’s continuous usage of a mobile application. Kasper and Abdelrahman [40] explained that people build trust and should have low risk perception to continuously use a technology. Moreover, Lallmahomed et al. [29] explained how trust is a key factor when determining intention to use an application. Therefore, the following were hypothesized:

**H9.**  *PR has a significant direct positive effect on intention to use the MorChana mobile application.*

**H10.**  *TR has a significant direct positive effect on intention to use the MorChana mobile application.*

U refers to an individual’s understanding of COVID-19 in relation to the causes, symptoms, prevention, and treatment of the COVID-19 pandemic [41]. Knowledge and understanding of a subject matter have been explained to be a key factor towards measuring an individual’s actions or behaviors, such as their intention [20]. Janmaimool [42] explained how an individual can be motivated to protect themselves when their health is at risk. Balkhy et al. [43] explained that when people know and understand an outbreak, they intend to prevent it. Therefore, it was hypothesized that:

**H11.**  *Understanding COVID-19 has a significant direct positive effect on intention to use the MorChana mobile application.*

IU is described as the extent of the intention to perform a particular action. Intention to use correlates positively with customers’ actual usage behavior [44]. Dehghani [45] explained how the intention to use and actual use have a significant effect when dealing with health-related goods and enabling technologies. Moreover, the study of Huang and Yang [46] explained how the intention to use and actual usage have a significant relationship when dealing with health-related technology. Thus, IU is the most obvious determinant of real-world information system behavior in pursuit of COVID -19. Therefore, it was hypothesized that:

**H12.**  *IU has a direct positive effect on the actual usage of the MorChana mobile application.*

## 3. Methodology

### 3.1. Participants

This study was approved by the Mapua University Research Ethics Committees and the Navaminda Kasatriyadhiraj Royal Thai Air Force Academy Research Ethics Committees (Document No: FM-RC-21-94). Collected via convenience sampling method, the participants considered were able to download and use the MorChana mobile application (Table 1).

This study considered data from 907 participants who voluntarily participated in this study. The survey was distributed online, and collected via different social media platforms from December 2021 to February 2022. The survey consisted of an introduction regarding the purpose and the consent form. Moreover, a question regarding the usage of MorChana was asked. Those who were not able to use the application could not proceed with the survey. A 5-point Likert scale was utilized throughout the whole survey.

The participants were 47.4% male and 52.6% female with ages ranging from 15–24 years old (37.3%), 25–34 years old (41.7%), to 35–44 years old (12.2%). The majority held a bachelor’s degree (60.09%), master’s degree (26.90%), or were high school graduates (11.91%). In addition, most of the respondents had a monthly salary/allowance of 10,001–20,000 THB (26.9%), 20,001–30,000 THB (28.4%), 30,001–40,000 THB (13.8%), less than 10,000 THB (15.7%), and 40,001–50,000 THB (11.4%). Lastly, 89.4% were not enrolled in COVID-19 insurance, and 10.6% were enrolled.

### 3.2. Questionnaire

Several indicators were considered in this study to measure the latent variables, such as performance expectancy, effort expectancy, social influence, facilitating conditions, hedonic motivation, habit, perceived risk, self-efficacy, privacy, trust, understanding COVID-19, intention to use the MorChana mobile application, and actual use behavior. A total of 53 questions adapted from several previous works were considered in this study. Presented in Table 2 are the constructs and measurement items.

The items of the questionnaire were adapted based on similar constructs from studies that utilized the same latent variable with mobile or technology application. Before the dissemination of the final questionnaire, an initial run considering 150 respondents was considered following the suggestion of Ong et al. [20]. A Cronbach’s alpha value of 8.053 was obtained from the preliminary run, which was deemed valid to be utilized in this study [41].

### 3.3. Structural Equation Modeling

Structural equation modeling (SEM) was considered in this study. SEM is a multivariate tool utilized to determine the causal relationships among the variables present in a model. This study considered a common method bias (CMB) SEM following the study of Prasetyo et al. [47]. Moreover, several studies considered SEM for determining factors affecting human behavior. An integration of UTAUT2 and PMT was considered by Jun et al. [48] regarding advanced driver assistance. In addition, Duarte and Pinho [49] considered SEM for evaluating mobile health adoption in Portugal. Alam et al. [16] focused on mobile health application adoption by integrating UTAUT2 and PMT in Bangladesh. They suggested that the model considered could be helpful in measuring factors affecting technology adoption and behavioral adoption. Thus, this study considered utilizing SEM to measure factors affecting actual usage behavior on the MorChana COVID-19 tracing mobile application required in Thailand upon entering any area or establishment.

## 4. Results

**Figure 3:**
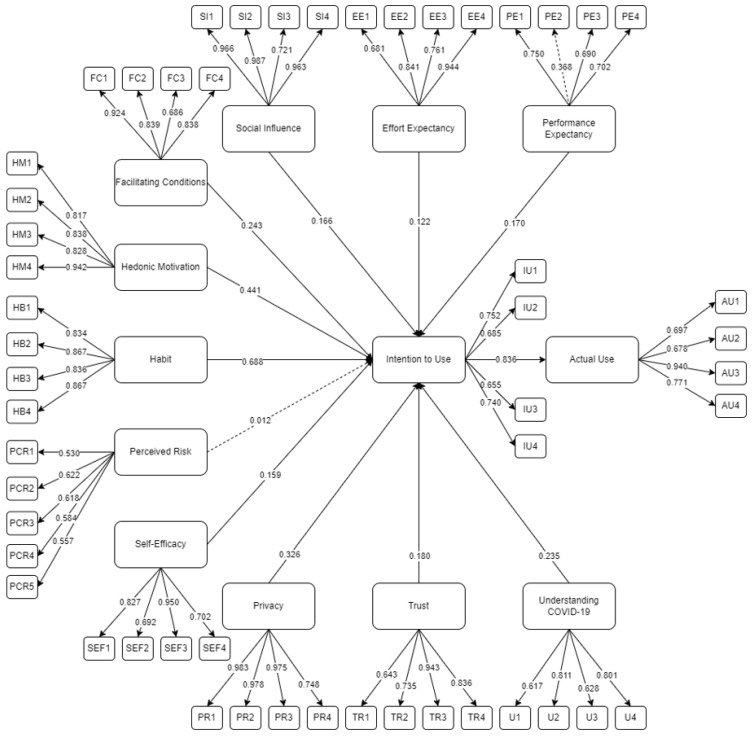
Initial SEM for intention to use the MorChana mobile application.

Presented in Figure 3 is the initial SEM run utilizing AMOS 25 following the study of Ong et al. [20]. From the initial SEM, perceived risk was seen to be insignificant (*p*-value > 0.05) and PE2 had a value less than 0.50. This was removed to enhance the model fit [50].

**Figure 4:**
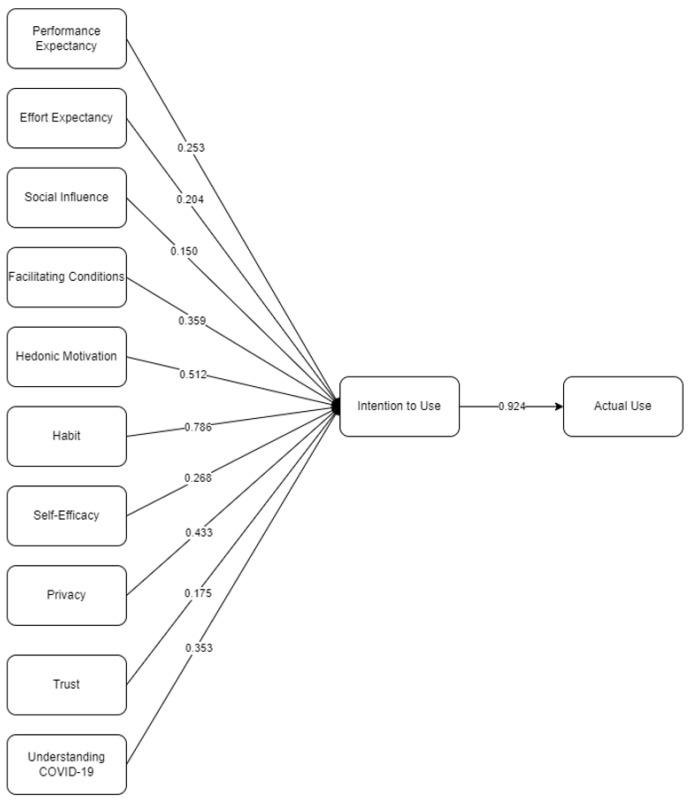
Final SEM for intention to use the MorChana mobile application.

The final SEM for the intention to use the MorChana mobile application is presented in Figure 3. Out of 12 hypotheses, 11 were considered significant as indicated by the solid lines. Presented as the dotted line is the insignificant hypothesis. Table 3 shows the descriptive statistics of the indicators, together with the initial and final factor loading. Based on the results, all final factor loadings were within the threshold of 0.50 [51]. After the removal of the insignificant latent variables, perceived risk (PCR *p*-value > 0.05) and low factor loading, PE2, the model was rerun. Presented in Figure 4 is the final SEM for measuring the intention to use the MorChana mobile application.

Table 4 presents the model fit for this study. Following the suggestion of Gefen et al. [52], the IFI, TLI, CFI, GFI, and AGFI should be greater than 0.80 to be considered acceptable. Moreover, Steiger [53] suggested that the RMSEA should be below 0.07; thus, the resulting final model is considered acceptable. Following Ong et al. [20], the common method bias (CMB) test using Harman’s single factor should be below 50%. This study presented a result of 20.235%, which indicates no CMB present.

To test the reliability and validity, Hair [50] suggested considering values of Cronbach’s alpha and composite reliability to be greater than 0.70. This study presented values greater than the threshold. In addition, the average variance extracted (AVE) should have values greater than 0.50. Thus, this model is considered to have been constructed with internal validity and reliability. In addition, testing of the multicollinearity of the variables was conducted, showing no multicollinearity. As suggested by Ong et al. [20], the variance inflation factor (VIF) should have a value less than 5.00 to indicate no multicollinearity. Presented in Table 5 are the results of the statistical analysis for validity.

Table 6 represents the direct, indirect, and total effects of the latent variables considered in this study. Based on the results, it could be seen that all latent variables remaining in the final model are significant, with values less than 0.05 [51,53]. Moreover, performing the Shapiro–Wilk test for normality showed the indicators to have values within the threshold, ±1.96 [54]. Thus, the presented final model is considered to be acceptable.

## 5. Discussion

This study considered the integration of PMT and UTAUT2, evaluated using SEM. Several factors, such as performance expectancy (PE), effort expectancy (EE), social influence (SI), facilitating conditions (FC), hedonic motivation (HM), and habit (HB) from UTAUT2; and perceived risk (PCR), self-efficacy (SEF), privacy (PR), trust (TR), and understanding of COVID-19 (U) from PMT, together with intention to use (IU), and actual usage (AU) were considered. With a highly acceptable model, several explanations and discussions could be applied from the results of this study.

Based on the results, IU had the highest significant direct effect on AU (β: 0.924; *p* = 0.003). From the indicators, it is suggested that people will continue utilizing MorChana, would install the mobile application if they changed phones, and would have support in utilizing the COVID-19 tracing application. This means that people will have a very high intention to continuously promote the utility of MorChana. Gelderblom et al. [55] presented how a positive intention would lead to the AU of a technology. Moreover, Pee et al. [56] showed that when an individual sees the advantage of using an application, the intention corresponds positively as well. This explains how people using MorChana are satisfied and would continuously patronize the utility of the mobile application. In accordance, Wu et al. [57] presented how intention is preceded by people’s satisfaction with using applications related to health. Their study showed that HB was also a significant factor.

Second, HB had a significant direct effect on IU (β: 0.786; *p* = 0.005), followed by HM (β: 0.512; *p* = 0.004). Based on the HB indicators, using the application became a habit, became a regular activity, and is present as a utility every day. HM indicators posit that MorChana is fun, enjoyable, entertaining, and pleasurable. This therefore presents an indirect effect of HB (β: 0.421; *p* = 0.010) and HM (β: 0.323; *p* = 0.003) on AU. Wu et al. [57] presented how HB is a strong determinant of satisfaction and continuous usage. They added that as their HB increases, the intention of a person using a health-related application also increases. Nikolopoulou et al. [58] presented how HM and HB are both latent variables that significantly affect an individual’s intention to use technology. Since using MorChana is required before entering any area in Thailand, it has become a habit for people to use the application every time. Moreover, Palau-Saumell et al. [59] explained how these are key indicators for people perceiving enjoyment and pleasure when incorporated into daily activities, which leads to continuous usage.

Fourth, PR (β: 0.433; *p* = 0.007) and TR (β: 0.175; *p* = 0.012) had a significant direct effect on IU. The people perceived that there is security for users’ information when utilizing the MorChana mobile application, privacy is prevalent in utilizing the application, and no GPS tracking is present. Moreover, Thais believed that MorChana is trustworthy, not opportunistic, expectations are met, and the content itself is reliable. This led to an indirect effect of PR (β: 0.215; *p* = 0.002) and TR (β: 0.054; *p* = 0.002) on AU. Choi et al. [60] explained how the security of technology enables the positive and negative perception of trust and risk, leading to the intention of usage. It was explained that people are highly sensitive when dealing with health-related information, and are inclined to consider their security [40,60]. Kapser and Abdelrahman [40] presented how risks in using an application should be reduced to increase trust that would have a positive intention of considering the usage of an application. Thus, it could be deduced that MorChana is highly trustworthy when it comes to securing information among users. which is why Thais would have the intention of using the mobile application.

Fifth, U (β: 0.353; *p* = 0.012) and SEF (β: 0.268; *p* = 0.017) had a significant direct effect on IU. From the indicators, people do understand the risks, symptoms, and health concerns of COVID-19 before using MorChana. This led to having SEF for MorChana, seeing that it is convenient, available, provides access to health services, and is easily utilized. This, in turn, caused an indirect effect on AU (U—β: 0.201; *p* = 0.008; SEF—β: 0.087; *p* = 0.008). When people understand the health risks present, they have self-efficacy to continuously utilize a technology [20]. Thakur [36] explained how people have positive self-efficacy when they have adapted to the system or technology at hand. This would lead to continuous intention to use an application.

Sixth, FC had a significant direct effect on IU (β: 0.359; *p* = 0.012). People have the necessary tools to use the MorChana mobile application, are knowledgeable in using the application, find it easy to use, and are able to ask if concerns arise. Subsequently, this had a positive indirect effect on AU (β: 0.186; *p* = 0.002). Lallmahomed et al. [29] explained that FC is one of the core latent variables for intention to use nowadays, due to the easy access to the internet. Alalwan et al. [61] also explained that people tend to utilize applications that are beneficial and available for utility. In the current generation, people consider the available resources for continuous usage [62].

SI was also seen to have a significant direct effect on IU (β: 0.150; *p* = 0.007) and an indirect effect on AU (β: 0.019; *p* = 0.005). People that are around and that are important to an individual affect their intention to use the mobile application, especially when these people around them also utilize MorChana. Palau-Saumell et al. [59] presented that when people around an individual suggest the application, there is also continuous utility due to the influence. Since people have been able to adjust to the current technological advancement, they would consider using the mobile application as well [63]. Ahmad and Khalid [62] also supported these findings, stating that people around the individual posit a positive IU.

Lastly, PE (β: 0.253; *p* = 0.011) and EE (β: 0.204; *p* = 0.012) had a significant direct effect on IU. In addition, PE (β: 0.152; *p* = 0.001) and EE (β: 0.180; *p* = 0.002) were seen to have an indirect effect on AU. People find MorChana useful in their daily lives, use it to help them prepare for COVID-19, and use it to help in assessing risk of COVID-19. Moreover, they find it easy to learn how to use MorChana, find that it is clear and understandable, experience easy utilization, and can be proficient. PE and EE were found to be significant factors affecting IU, based on several studies [57,58,64]. Wu et al. [56] explained that when people find technology useful for health-related effects and daily activities, together with easy utility of the application, they have positive intentions of continuous usage. Supported by Nikolopoulou et al. [58], with positive PE and EE, people will find the application valuable and continuously use the technology.

Overall, the actual usage of a health-related mobile application such as MorChana was deemed to be a pleasant experience, to have ease of utility, and to have been used as part of the daily activity of people. In addition, it was deemed important that this application has helped people in protecting themselves against COVID-19. There is a perception that MorChana helps in mitigating the risk of COVID-19 exposure. Thus, continuous usage of the application would be seen even in the future, due to its health benefits and protective effects.

### 5.1. Theoretical Contribution

Common studies have been considered to extend the measures of technology and health [22,31,58,64]. This study was able to promote the integration of PMT and UTAUT2. From the model, only one latent variable (perceived risk) was seen to be insignificant. It could therefore be deduced that this model can holistically measure the fear and coping appraisal of people, their behavior, and the actual usage of health-related technologies.

When trust and privacy assurance in using an application are instigated in people, there is a positive influence towards continuous patronage. Since health-related information is highly protected, the behavior of people would lead to the acceptance of the mobile application, leading to actual use. Based on the findings, people will adapt to the system when they utilize it every day, perceive it to be easy to use, find it beneficial for health, experience less effort in navigation, know other people utilizing the application as well, and find that it has the ability to inform and protect. Thus, health-related mobile applications could refer to these factors for the enhancement of continuous patronage.

### 5.2. Practical Implications

Based on the findings of this study, MorChana was seen to be highly considered among people in Thailand. With the strict implementation of the application, it has led to making the utility a habit, which in turn has led to actual usage. Moreover, when people see the benefit, and trust is developed, people will continuously use the system even in the future. Developers could capitalize on the findings of this study. They could consider a health-related mobile application that is easily navigated, and easily understood, making it fun and enjoyable, informative, and secure. This will result in a positive outcome for people to download and consider actual usage, even in the future.

Despite the plan to mitigate and reduce the spread of the COVID-19 virus, Thailand is still the second most highly infected Asian country [14]. As a result, people are still required to utilize the mobile application, MorChana. The results of this study would therefore help in promoting the utility of MorChana. It could be highlighted that when people know the benefit, have a grasp of the application, and have ease of use, then continuous patronage will be seen. The measurements and constructs found in this study could also be extended and adapted to develop the system for other uses. Other mobile applications may also consider the findings of this study for the purposes of adaptation and development worldwide.

### 5.3. Limitations and Future Research

Despite the significant findings of this study, several limitations and extensions may still be considered. This study used an online self-administered survey, which may be a limitation due to the consideration of the latent variables coming from factors under PMT and UTAUT2. Interviews could be considered instead. This would help assess the findings of this study, and even create the extension and development of latent constructs. Second, the older generation was significantly scarce in this study. Ong et al. [65] explained how younger generations would be the ones available to answer an online self-administered survey, since they are mostly online due to classes and work. Thus, after the lockdown, it could be recommended to consider the older generation, which may result in other relevant findings. Lastly, the incorporation of machine learning algorithms such as neural networks or random forest classifiers may be performed. With that, the classification of factors affecting actual usage may be deduced. Overall, clustering the constructs or demographics of this study may present other findings, which were not seen from SEM.

## 6. Conclusions

Since the start of the pandemic in 2020, many countries, together with the World Health Organization, have tried several mitigation strategies to control the spread of COVID-19. One of the developments was the consideration of COVID-19 tracing applications, which have been utilized worldwide, in a different form for every country. One of which is the MorChana COVID-19 tracing mobile application of Thailand. Despite the availability of several studies regarding the adaptation and utility of tracing applications, no studies were available dealing with the application of contact tracing in Thailand.

Collecting a total of 907 valid responses, this study aimed to evaluate the MorChana COVID-19 tracing mobile application through the integration of PMT and UTAUT2. Several factors such as performance expectancy (PE), effort expectancy (EE), social influence (SI), facilitating conditions (FC), hedonic motivation (HM), and habit (HB) from UTAUT2; and perceived risk (PCR), self-efficacy (SEF), privacy (PR), trust (TR), and understanding of COVID-19 (U) from PMT, together with intention to use (IU) and actual usage (AU), were considered in this study, evaluated through structural equation modeling. The results show that IU was the most significant factor affecting AU. This is because people were able to see the health advantages of the mobile application.

Following this, HB, HM, PR, FC, U, SEF, PE, EE, TR, and SI were also seen to be significant factors. People find the application to be a pleasant experience, have ease of utility, and have used the application as part of their daily activity. In addition, it was deemed important that this application has helped people in protecting themselves against COVID-19. This has motivated people to use the MorChana application. Overall, the integration of PMT and UTATU2 could be interpreted as a model that can holistically measure the intention and actual usage of a health-related mobile application. The integrated framework can be applied and extended to determine the factors affecting COVID-19 tracing applications in other countries. Moreover, the findings of this study could be applied in other health-related mobile applications worldwide [66,67,68,69].

## Figures and Tables

**Table 1:** Descriptive variables of survey (n = 907).

Characteristics	Category	N	%
Gender	Male	430	47.4
	Female	477	52.6
Age	15–24	338	37.3
	25–34	378	41.7
	35–44	111	12.2
	45–54	55	6.1
	55–64	22	2.4
	More than 64	3	0.3
Education Level	High school graduate	108	11.91
	Bachelor’s degree	545	60.09
	Master’s degree	244	26.90
	Doctoral degree	10	1.100
Monthly Salary/Allowance	Less than 10,000 THB	142	15.7
	10,001–20,000 THB	244	26.9
	20,001–30,000 THB	258	28.4
	30,001–40,000 THB	125	13.8
	40,001–50,000 THB	103	11.4
	More than 50,000 THB	35	3.9
Enrolled in COVID-19 insurance?	Yes	96	10.6
	No	811	89.4

**Table 2:** Construct and measurement items.

Constructs	Item	Measurement	References
Performance Expectancy	PE1	I find MorChana apps useful in my life.	Alam et al. [16]
	PE2	Using MorChana apps increases my prevention of COVID-19.	Alam et al. [16]
	PE3	Using MorChana apps helps me prepare for COVID-19 prevention more easily.	Venkatesh et al. [15]
	PE4	Using MorChana apps helps me assess the risk of COVID-19 in my daily life.	Venkatesh et al. [15]
Effort Expectancy	EE1	Learning to use MorChana apps is easy for me.	Venkatesh et al. [15]
	EE2	My interaction with MorChana apps is clear and understandable.	Alam et al. [16]
	EE3	I find MorChana apps easy to use.	Venkatesh et al. [15]
	EE4	I find it easy to use the MorChana apps proficiently.	Alam et al. [16]
Social Influence	SI1	People who are important to me think I should use MorChana apps.	Alam et al. [16]
	SI2	People who influence my behavior think I should use MorChana apps.	Alam et al. [16]
	SI3	People whose opinions I value prefer that I use MorChana apps.	Venkatesh et al. [15]
	SI4	People who use MorChana apps have more prestige in my society.	
Facilitating Conditions	FC1	I have the necessary resources to use MorChana apps.	Venkatesh et al. [15]
	FC2	I have the necessary knowledge and skills to use MorChana apps.	Alam et al. [16]
	FC3	I can get help from others if I have difficulty using MorChana apps.	Venkatesh et al. [15]
	FC4	MorChana apps are easy to use with my mobile phone.	Alam et al. [16]
Hedonic Motivation	HM1	Using MorChana apps is fun.	Alam et al. [16]
	HM2	Using MorChana apps is enjoyable.	Venkatesh et al. [15]
	HM3	Using MorChana apps is entertaining.	Alam et al. [16]
	HM4	Using MorChana apps is pleasurable.	Alam et al. [16]
Habit	HB1	Using MorChana apps has become a habit for me.	Venkatesh et al. [15]
	HB2	I am addicted to using MorChana apps.	Alam et al. [16]
	HB3	Using MorChana apps has been a regular activity for me.	Venkatesh et al. [15]
	HB4	Using MorChana apps has become a natural activity for me.	Alam et al. [16]
Perceive Risk	PCR1	Using MorChana apps helps me assess symptoms of COVID-19.	Ong et al. [20]
	PCR2	Using MorChana apps helps me to identify the risk area for COVID-19.	Ong et al. [20]
	PCR3	Using MorChana apps helps me identify who is at risk of COVID-19.	Ong et al. [20]
	PCR4	Using MorChana apps still makes you an at-risk person of COVID-19.	Ong et al. [20]
	PCR5	Using MorChana apps helps warn other users who visited the same place as the infected person at the same time.	
Self-Efficacy	SEF1	It is convenient for me to use MorChana apps.	Alam et al. [16]
	SEF2	I am able to use MorChana apps.	Alam et al. [16]
	SEF3	I would be able to use MorChana apps to access health services if there was no one around to tell me what to do.	Alam et al. [16]
	SEF4	I could access COVID-tracking system using MorChana apps if I had never used one before.	Alam et al. [16]
Privacy	PR1	I believe that the privacy of users of MorChana apps is protected.	Alam et al. [16]
	PR2	I believe that personal information stored in MorChana apps system is secure.	Alam et al. [16]
	PR3	I believe that MorChana apps keeps participants’ information secure.	Alam et al. [16]
	PR4	I believe that MorChana apps do not use GPS or track mobile phone location.	Alam et al. [16]
Trust	TR1	I know that MorChana apps is trustworthy.	Alam et al. [16]
	TR2	I know that MorChana Apps is not opportunistic.	Alam et al. [16]
	TR3	I know that MorChana Apps keeps its promises to its users.	Alam et al. [16]
	TR4	The content of MorChana apps is reliable.	Alam et al. [16]
Understanding of COVID-19	U1	I do understand the distribution of COVID-19 before using MorChana apps.	Prasetyo et al. [41]
	U2	I do understand the incubation period of COVID-19 before using MorChana apps.	Prasetyo et al. [41]
	U3	I do understand the symptoms of COVID-19 before using MorChana apps.	Prasetyo et al. [41]
	U4	I do understand how to prevent COVID-19 before I use MorChana apps.	Prasetyo et al. [41]
Intention to use MorChana application	IU1	I intend to continue using MorChana apps in the future.	Venkatesh et al. [15]
	IU2	I will always try to use MorChana apps in my daily life.	Venkatesh et al. [15]
	IU3	I plan to continue to use MorChana apps frequently.	Venkatesh et al. [15]
	IU4	I would install MorChana apps when I get a new mobile phone.	Venkatesh et al. [15]
Actual Usage Behavior	AU1	MorChana apps are a pleasant experience.	Prasetyo et al. [41]
	AU2	I really use MorChana apps to protect my health.	Alam et al. [16]
	AU3	I spend a lot of time using MorChana apps.	Venkatesh et al. [15]
	AU4	I use MorChana apps on a regular basis.	Prasetyo et al. [41]

**Table 3:** Indicators from statistical analysis.

Variable	Item	Mean	StD	Factor Loading	
				Initial	Final
Performance Expectancy	PE1	4.1422	1.11514	0.750	0.733
	PE2	4.0573	1.11235	0.368	-
	PE3	4.1312	1.13318	0.690	0.693
	PE4	3.5877	1.45187	0.702	0.704
Understanding COVID-19	U1	4.3495	0.86265	0.617	0.617
	U2	4.2194	0.91669	0.811	0.809
	U3	4.2966	0.87472	0.628	0.629
	U4	4.1125	1.02858	0.801	0.802
Trust	PT1	3.6604	1.38957	0.643	0.643
	PT2	3.7365	1.34952	0.735	0.735
	PT3	4.2095	1.01653	0.943	0.944
	PT4	4.3230	0.91005	0.836	0.748
Perceived Risk	PCR1	3.5777	1.48475	0.983	0.983
	PCR2	3.7630	1.36982	0.978	0.978
	PCR3	3.7277	1.37051	0.975	0.974
	PCR4	3.5193	1.49997	0.748	0.674
Self-Efficacy	SEF1	4.1345	1.03557	0.827	0.827
	SEF2	4.2183	1.03833	0.692	0.692
	SEF3	3.5215	1.48217	0.950	0.949
	SEF4	4.1621	1.10246	0.702	0.703
Habit	HB1	3.3462	1.58264	0.834	0.817
	HB2	3.2679	1.69791	0.867	0.833
	HB3	3.2900	1.59841	0.836	0.867
	HB4	3.2900	1.65939	0.867	0.836
Hedonic Motivation	HM1	3.3374	1.54504	0.817	0.838
	HM2	3.4465	1.52614	0.838	0.828
	HM3	3.3506	1.58863	0.828	0.642
	HM4	3.8875	1.13859	0.942	0.942
Facilitating Conditions	FC1	3.7398	1.30485	0.924	0.923
	FC2	3.2834	1.68036	0.839	0.623
	FC3	4.0959	1.08708	0.686	0.686
	FC4	4.0276	1.10103	0.838	0.838
Social Influence	SI1	3.8148	1.34742	0.966	0.966
	SI2	3.7552	1.34035	0.987	0.987
	SI3	3.6759	1.40273	0.721	0.663
	SI4	3.6615	1.39973	0.963	0.963
Effort Expectancy	EE1	4.1698	1.00871	0.681	0.681
	EE2	3.9327	1.22650	0.841	0.841
	EE3	3.9857	1.20742	0.761	0.761
	EE4	3.9338	1.22431	0.944	0.945
Intention to Use	IU1	3.6549	1.35888	0.752	0.753
	IU2	4.0176	1.09872	0.685	0.686
	IU3	4.0386	1.08046	0.655	0.658
	IU4	3.7343	1.34335	0.740	0.740
Actual Use	AU1	3.7817	1.30831	0.697	0.697
	AU2	3.8886	1.17543	0.678	0.678
	AU3	3.1709	1.68637	0.940	0.941
	AU4	3.4388	1.58196	0.771	0.772

**Table 4:** Model fit.

Goodness of Fit Measures of SEM	Parameter Estimates	Minimum Cut-Off	Suggested by
Incremental Fit Index (IFI)	0.901	>0.80	Gefen et al. [52]
Tucker–Lewis Index (TLI)	0.893	>0.80	Gefen et al. [52]
Comparative Fit Index (CFI)	0.900	>0.80	Gefen et al. [52]
Goodness of Fit Index (GFI)	0.837	>0.80	Gefen et al. [52]
Adjusted Goodness of Fit Index (AGFI)	0.859	>0.80	Gefen et al. [52]
Root Mean Square Error (RMSEA)	0.062	<0.07	Steiger [53]

**Table 5:** Composite reliability and validity.

Factor	Cronbach’s α	Composite Reliability (CR)	Average Variance Extracted (AVE)	Variance Inflation Factor (VIF)
Performance Expectancy	0.753	0.791	0.504	2.166
Effort Expectancy	0.885	0.788	0.661	2.071
Social Influence	0.946	0.779	0.819	3.323
Facilitating Conditions	0.856	0.784	0.603	3.111
Hedonic Motivation	0.889	0.861	0.672	4.312
Habit	0.904	0.913	0.703	4.576
Self-Efficacy	0.875	0.757	0.639	2.160
Perceived Risk	0.951	0.789	0.831	1.161
Trust	0.855	0.752	0.601	2.605
Understanding COVID-19	0.851	0.713	0.519	1.341
Intention to Use	0.802	0.727	0.505	3.084
Actual Use	0.858	0.834	0.607	-

**Table 6:** Direct, indirect, and total effects.

No	Variable	Direct Effect	*p*-Value	Indirect Effect	*p*-Value	Total Effect	*p*-Value
1	PE → IU	0.253	0.011	-	-	0.253	0.011
2	EE → IU	0.204	0.012	-	-	0.204	0.012
3	SI → IU	0.150	0.007	-	-	0.150	0.007
4	FC → IU	0.359	0.012	-	-	0.359	0.012
5	HM → IU	0.512	0.004	-	-	0.512	0.004
6	HB → IU	0.786	0.005	-	-	0.786	0.005
7	SEF → IU	0.268	0.017	-	-	0.268	0.017
8	PR → IU	0.433	0.007	-	-	0.433	0.007
9	TR → IU	0.175	0.004	-	-	0.175	0.004
10	U → IU	0.353	0.012	-	-	0.353	0.012
11	IU → AU	0.924	0.003	-	-	0.924	0.003
12	PE → AU	-	-	0.152	0.001	0.152	0.001
13	EE → AU	-	-	0.180	0.002	0.180	0.002
14	SI → AU	-	-	0.019	0.005	0.019	0.005
15	FC → AU	-	-	0.186	0.002	0.186	0.002
16	HM → AU	-	-	0.323	0.003	0.323	0.003
17	HB → AU	-	-	0.421	0.010	0.421	0.010
18	SEF → AU	-	-	0.087	0.008	0.087	0.008
19	PR → AU	-	-	0.215	0.002	0.215	0.002
20	TR → AU	-	-	0.054	0.002	0.054	0.002
21	U → AU	-	-	0.201	0.008	0.201	0.008

## Data Availability

The data presented in this study are available on request from the corresponding author.
